# Risk factors and outcome of asparaginase-associated pancreatitis in pediatric acute lymphoblastic leukemia

**DOI:** 10.3389/fonc.2025.1606261

**Published:** 2025-06-26

**Authors:** Seham Hassan, Sonia Ahmed, Nesreen Ali, Abeer Mokhles, Iman Zaky, Hala Reda, Sarah H. Youssef, Dina Hammad, Omneya Hassanain, Iman Sidhom

**Affiliations:** ^1^ Pediatric Oncology Department, Children’s Cancer Hospital Egypt, Cairo, Egypt; ^2^ Pediatric Oncology Department, National Cancer Institute, Cairo University, and Children’s Cancer Hospital Egypt, Cairo, Egypt; ^3^ Clinical Oncology Department, Beni-Suef University, Egypt and Children’s Cancer Hospital Egypt, Cairo, Egypt; ^4^ Radiology Department, National Cancer Institute, Cairo University, and Children’s Cancer Hospital Egypt, Cairo, Egypt; ^5^ Clinical Pathology Department, National Cancer Institute, Cairo University, and Children’s Cancer Hospital Egypt, Cairo, Egypt; ^6^ Department of Pharmaceutical Services, Children’s Cancer Hospital Egypt, Cairo, Egypt; ^7^ Institute of Global Health and Human Ecology, The American University in Cairo (AUC), Cairo, Egypt; ^8^ Clinical Research Department, Children’s Cancer Hospital Egypt (57357), Cairo, Egypt

**Keywords:** acute lymphoblastic leukemia, pancreatitis, asparaginase, risk factors, pediatric, low and middle-income countries

## Abstract

**Background:**

Asparaginase-associated pancreatitis (AAP) poses a significant challenge in pediatric patients with acute lymphoblastic leukemia (ALL), with its severity ranging from mild cases to potentially life-threatening conditions.

**Aim:**

To study the incidence, risk factors of AAP, and its impact on outcome in pediatric ALL patients in a large pediatric oncology hospital in a low-and middle-income country.

**Patient and methods:**

This retrospective study included 1804 pediatric patients newly diagnosed with ALL at a single tertiary care center from June 2012 to December 2017. They were treated with ALL protocol adopted from St Jude total study XV including native E. coli L-asparaginase.

**Results:**

Sixty-three (3.5%) patients experienced AAP. Age ≥10 years at diagnosis, initial white blood cell count (WBC) ≥50x10^9^/L, and standard/high-risk treatment regimen were significantly associated with developing AAP. By multivariate analysis, age ≥10 years and high-dose asparaginase regimens remained significant risk factors for AAP. Mild/moderate AAP was reported in 47 (75%) patients without associated mortality, however, 6/16 (37.5%) patients with severe pancreatitis died. Asparaginase was re-challenged in 39/63 (62%) patients of whom 12 patients (30.8%) experienced recurrent AAP without mortality. Patients who were not re-exposed to asparaginase had a relapse rate of 37.5% compared to 23% for those who were re-challenged. The 5-year event-free-survival (EFS) and cumulative incidence of relapse (CIR) were 63.5% and 24.3%; respectively; for patients with AAP compared to 77% and 14.4% for those without AAP (P=0.01, P=0.02; respectively). However, AAP lost its significant impact when adjusted to other factors including age, WBC, immunophenotype, and ALL risk stratification (EFS: HR1.32; 95%CI, 0.88-1.98; P=0.17 and CIR: HR1.44; 95%CI, 0.86-2.4; *P=0*.16).

**Conclusion:**

Older age and high-dose asparaginase regimens are independent risk factors of AAP. The decision to re-challenge asparaginase should be carefully considered balancing the risk of recurrent pancreatitis against the potential risk of leukemic relapse.

## 1 Introduction

Over the past five decades, the cure rates for pediatric acute lymphoblastic leukemia (ALL) have improved dramatically, with over 90% of children now experiencing long-term survival. The primary factor contributing to this significant improvement is the integration of multiagent chemotherapy regimens. Asparaginase (ASNase) is the most widely used chemotherapeutic agent introduced into the multi-agent backbone therapy for ALL (1). It is a crucial drug in inducing remission in pediatric ALL, by promoting the hydrolysis of extracellular asparagine, which is essential for the growth of leukemic lymphoblasts. This process also inhibits the synthesis of leukemic lymphoblast protein, resulting in apoptosis with minimal myelosuppression (2, 3).

Currently, there are three principal formulations of ASNase available in the market. Two originate from Escherichia coli (E. coli): the native L-ASNase and its pegylated variants, (PEG-ASNase, and Calaspargase pegol). The third formulation is the Erwinase, which is derived from Erwinia Chrysathemi. These formulations have distinctive pharmacokinetic, pharmacodynamic, and immunogenic characteristics (4–6). In recent years, PEG-ASNase has become the preferred choice in high-income countries, resulting in decreased demand for non-pegylated formulations. However, due to the high cost of PEG-ASNase, the non-pegylated form is still frequently used to treat ALL in low-and middle-income countries (LMICs) (7). Although ASNase is crucial in treating pediatric ALL, its frequent administration can result in various adverse effects. Asparaginase-associated pancreatitis (AAP) is a notable complication, occurring in up to 18% of children and young adults undergoing treatment for ALL (8–11). Despite low mortality, AAP frequently results in the premature discontinuation of ASNase therapy, which may increase the risk of leukemia relapse (12). Additionally, re-exposure to ASNase following AAP has been linked to a nearly 50% increased risk of recurrent pancreatitis, which is the main cause of ASNase truncation in the affected children (2).

Given the significant impact of AAP on ASNase therapy and its potential effects on treatment outcomes, this study was conducted to assess the incidence and potential risk factors associated with AAP, as well as to evaluate its impact on the outcomes of ALL. It focuses on children receiving chemotherapy that includes native L-ASNase, a widely used and affordable formulation in LMICs, aiming to provide valuable insights into the burden of AAP in resource-limited settings.

## 2 Patients and methods

This retrospective study included 1,804 pediatric patients, all under the age of 18 years who were diagnosed with ALL at Children’s Cancer Hospital Egypt (CCHE) between June 2012 and December 2017. The treatment approach was based on a risk-directed protocol adapted from the St Jude Total Therapy Study XV (13).

### 2.1 Patient risk stratification

Our patients were classified into four risk groups, very low risk (VLR), low risk (LR), standard risk (SR), and high risk (HR) based on their baseline age, white blood cell count (WBC), ALL immunophenotype (IPT), DNA index, genetic profiles, CNS status, presence or absence of overt testicular leukemia, and response to therapy assessed by levels of minimal residual disease (MRD) measured by flow cytometry. The criteria for each group are presented in **Supplementary Table S1**.

### 2.2 Application of ASNase in CCHE-adopted St. Jude ALL total-XV protocol

Escherichia coli L- ASNase (Medac- ASNase) was the standard asparaginase preparation, administered as intramuscular injections. The dosage and frequency of ASNase injections vary based on the patient’s risk assessment.

Notably, both SR and HR groups received the same combination of chemotherapeutic agents, as outlined in the total study XV during induction, consolidation, reinduction, and continuation therapy. However, high-risk patients were scheduled to receive an additional re-intensification cycle after consolidation therapy or one cycle of re-intensification post-reinduction I for those with MRD ≥0.1 at week 7 of continuation therapy.

#### 2.2.1 Remission induction therapy

L-ASNase was administered three times weekly at a dose of 5000 IU/m^2^ per dose for a total of six doses. Patients with MRD ≥1% on day 15 remission induction received additional three doses of L- ASNase.

#### 2.2.2 Continuation and reinduction therapy

For SR/HR patients, L-ASNase was administered at a dose of 12500 IU/m^2^ once weekly for 19 weeks during the continuation therapy, including reinduction I (weeks 7-9) and reinduction II (weeks17-19), while LR patients received L-ASNase, 5000 IU/m^2^ per dose, every other day (3 doses/week) during the reinduction I and II (weeks: 7–9 and 17–19) for a total of 18 doses. VLR patients received four doses of L-ASNase, 12500 IU/m^2^ per dose, on days 1, 8, 15, and 22, during the reinduction phase.

#### 2.2.3 Reintensification therapy

HR patients were scheduled to receive re-intensification treatment with L-ASNase 12500 IU/m^2^ administered as a single dose. Timings and doses of L-ASNase administration in the total therapy study XV are illustrated in **Supplementary Figure S1**.

### 2.3 AAP diagnostic criteria

Acute Pancreatitis (AP) was diagnosed using the consensus criteria established by the Ponte di Legno Toxicity Working Group (PTWG) (8), which require at least two of the following three criteria to be fulfilled: severe abdominal pain suggestive of pancreatitis; elevated serum amylase or lipase levels ≥3 times the upper normal limit; and characteristic imaging features consistent with AP.

### 2.4 Criteria of severity in AAP

The Common Terminology Criteria for Adverse Events (CTCAE, version 5) was utilized for AP grading and the patients were classified into two groups based on the severity of AAP. Mild/moderate AAP comprised Grade 3 pancreatitis defined as severe pain or vomiting, necessitating medical intervention (e.g., analgesia, or nutritional support). Severe AAP constituted Grade 4 pancreatitis defined as life-threatening consequences or urgent intervention required (14).

Hypovolemic shock, systemic inflammatory response syndrome (SIRS), multi-organ failure (MOF), necrotizing pancreatitis, and acute pancreatic pseudocysts were deemed criteria for severe AAP. Mortality related to acute pancreatitis was defined as death within 14 days of the condition’s onset (15, 16).

### 2.5 Data collection

The patients´ electronic medical records were revised retrospectively for the data that confirmed the patient’s risk category, diagnosis of acute pancreatitis, including the presenting symptoms, amylase and lipase levels, abdominal contrast-enhanced CT scan. The total number and doses of L-ASNase administered before the occurrence of AAP, and after ASNase rechallenge, as well as the number of days between the last L-ASNase administration and the onset of AAP, were calculated. Local and systemic consequences of AAP were retrieved. The primary disease outcome of all patients was recorded.

### 2.6 Statistical analysis

Analyses focused on baseline characteristics, cumulative asparaginase dose, clinical outcomes, and prognosis. Continuous variables were summarized as mean with standard deviation (SD) or median with range and compared using the Kruskal-Wallis test. Categorical variables were summarized as frequencies and percentages, and compared using chi-square or Fisher’s exact test, as appropriate. A p-value of <0.05 was considered statistically significant. Statistical analyses were conducted using R software (version 4.2.1).

Event-free survival (EFS), overall survival (OS), and cumulative incidence of relapse (CIR) were evaluated across the cohort and compared between pancreatitis and non-pancreatitis groups.

EFS was defined as the time from treatment initiation to the first event (death in induction, relapse, second malignancy, refractory disease, or death in CR) or the date of last contact. OS was defined as the time from treatment initiation to death from any cause or to censoring at the date of the last follow-up for surviving patients.

The Kaplan-Meier method was used to estimate OS and EFS. Cox proportional hazards regression was used to evaluate the association between clinical covariates and survival outcomes, with hazard ratios (HR) and 95% confidence intervals (CI) calculated. Both univariate and multivariate Cox models were applied. For the multivariate analysis, we calculated events‐per‐variable (EPV) following Peduzzi et al (17), who recommend a minimum of 10 EPV for stable estimates. With 377 OS events and 457 EFS events in our cohort, the overall EPV for five covariates exceeded 10; however, the pancreatitis covariate alone yielded fewer than 10 events, risking sparse‐data bias. To mitigate this, adjusted HRs for pancreatitis were estimated using Firth’s penalized Cox regression (R package coxphf).

Potential multicollinearity among continuous covariates (age at diagnosis and baseline WBC level) and the categorical treatment-regimen variable was evaluated by computing variance inflation factors (VIF) with the car package in R. All VIFs were <2, and pairwise correlation coefficients were <0.3, indicating no significant multicollinearity.

Cumulative incidence functions (CIF) were used to estimate the cumulative incidence of relapse (CIR) accounting for death as a competing event. The Fine and Gray sub-distribution hazards model was applied to assess the impact of covariates on CIR, with sub-distribution hazard ratios (sHR) and 95% confidence intervals reported.

## 3 Results

### 3.1 Patients’ characteristics

Of the 1804 patients diagnosed with ALL over 5 years, sixty-four patients developed acute pancreatitis. One patient developed pancreatitis before asparaginase treatment initiation and was excluded from the study. Thus, the incidence of AAP was reported in sixty-three patients constituting 3.5%. **Table 1** summarizes the baseline and underlying disease features of patients who developed AAP.

**Table 1:** Baseline and underlying disease features of patients with AAP.

Characteristics	AAP patients (n=63) N (%)
Age (Years)	
Median [Min, Max]	8.81 [1.08, 17.8]
<10	39 (61.9%)
≥ 10	24 (38.1%)
Gender	
Male	39 (61.9%)
Female	24 (38.1%)
Baseline WBC	
**<**50×10^9^/L	43 (68.3%)
≥50×10^9^/L	20 (31.7%)
Immunophenotype (IPT)	
B-Cell	46 (73.0%)
T- Cell	17 (27.0%)
Non-random Fusion Genes in (B-cell, n=46)	
ETV6-RUNX1	7 (11.1%)
TCF3-PBX1	5 (7.9%)
BCR-ABL1	2 (3.2%)
No aberration	32 (50.8%)
Risk Group	
VLR	2 (3.2%)
LR	12 (19.1%)
SR	42 (66.7%)
HR	7 (11%)
AAP Chemotherapy Cycle	
Remission induction	35 (55.6%)
LR Reinduction therapy	6 (9.5%)
SR/HR Continuation (weeks 1 to 19)	21 (33.3%)
Reintensification	1 (1.6%)
AAP severity	
Mild/Moderate	47 (74.6%)
Severe	16 (25.4%)

AAP, asparaginase- associated pancreatitis; WBC, white blood cell count; SR/HR, standard-risk/high-risk; LR, low-risk; VLR, very low-risk.

### 3.2 Clinical features, laboratory and imaging findings

AAP-related symptoms occurred at a median interval of 4 days (range: 1–17 days) from the last ASNase administration. The median number of L- ASNase injections before AAP onset was 9 (range:1-28) with a median cumulative dose of 45000 IU/m^2^ (range: 5000–282,500 IU/m^2^). AAP was most frequently observed during the remission induction (55.6%; n=35), followed by 33.3% (n=21), 9.5% (n=6), and 1.6% (n=1) during the SR/HR continuation, LR reinduction and HR re-intensification phases, respectively.

Besides abdominal pain, the most prevalent symptom among all patients, vomiting was reported in 44 patients, fever in 27, and diarrhea in 2 patients. Serum amylase was assessed in 62 patients, of whom 26 patients (42%) had an elevated level ≥3 times the upper normal limit with a median peak value of 351 U/L (range: 95.0–3040 U/L). The Serum amylase returned to normal levels within a median of 5 days (range: 1–65 days). Additionally, serum lipase level was tested in 56 patients, 51 patients (91%) experienced three times higher levels than normal, with a median peak value of 663 U/L (range: 82–2710 U/L). Serum lipase returned to normal levels within a median of 10 days (range: 2–50 days). However, serum amylase and lipase levels remained high in one patient and were associated with recurrent abdominal pain for several months due to the persistence of chronic pancreatic pseudocysts.

Contrast-enhanced abdominal CT scan was the primary imaging technique performed in all patients. Forty-one patients (65%) had characteristic imaging features consistent with AP, the remaining 22 patients had negative radiological findings and were diagnosed based on their clinical symptoms and elevated serum amylase or lipase levels.

### 3.3 Risk factors associated with AAP

Age ≥10 years, WBC ≥50×10^9^/L, and high-dose asparaginase regimens for SR/HR patients were identified as risk factors for developing pancreatitis, however, only age and SR/HR regimen were associated with higher odds of developing pancreatitis on multivariate analysis (**Table 2**).

**Table 2:** Asparaginase-associated pancreatitis in relation to patient and disease characteristics.

Characteristics	Non-AAP (N=1740)	AAP (N=63)	Univariate analysis P-value	Multivariate analysis P-value	Odds ratio (95%CI)
Age (Years)					
Mean (SD)	6.47 (4.16)	8.36 (4.57)	<0.001	0.04	1.8(1.02-3.13)
Median [Min, Max]	5.22 [0.300, 18.0]	8.81 [1.08, 17.8]			
<10	1391 (79.9%)	39 (61.9%)	0.001		
≥ 10	349 (20.1%)	24 (38.1%)			
Gender					
Male	1024 (58.9%)	39 (61.9%)	0.696		
Female	716 (41.1%)	24 (38.1%)			
Immunophenotype					
B- Cell	1430 (82.2%)	46 (73.0%)	0.068	0.97	0.99 (0.51-1.84)
T- Cell	310 (17.8%)	17 (27.0%)			
Baseline WBC					
Mean (SD)	43.0 (83.8)	60.5 (97.8)	0.042	0.25	1.42(0.77-2.52)
Median [Min, Max]	10.8 [0.30, 845]	16.2 [1.3, 437.6]			
<50×10^9^/L	1394 (80.1%)	43 (68.2%)	0.025		
≥50×10^9^/L	346 (19.9%)	20 (31.8%)			
Treatment Regimen					
SR/HR	968(55.6%)	49 (77.8%)	0.001	0.16 0.04	0.6 (0.29-1.2) 0.22 (0.03-0.76)
LR	514 (29.5%)	12 (19.0%)			
VLR	258 (14.8%)	2 (3.2%)			

AAP, asparaginase- associated pancreatitis; WBC, white blood cell count; SR/HR, standard-risk/high-risk; LR, low-risk; VLR, very low-risk.

### 3.4 AAP severity and associated risk factors

Of the 63 patients diagnosed with AAP, 47 patients (74.6%) had mild/moderate AAP. Among the 16 patients with severe AAP, four patients had acute necrotizing pancreatitis, and three patients developed acute pancreatic pseudocysts. Of these, two required cyst drainage via endoscopic cystogastrostomy to relieve persistent pain and compression-related symptoms. Additionally, eleven patients experienced hypovolemic shock, with eight requiring admission to the pediatric intensive care unit (PICU). Two patients progressed to multiorgan failure, necessitating hemodialysis and mechanical ventilation. The median duration of PICU stay was 7.5 days (range: 2–24 days). Notably, none of the patients with mild/moderate AAP required PICU admission. Patients aged ≥10 years were more likely to develop severe AAP compared to those who were younger than 10 years (P=0.003). No additional factors were identified to be associated with the severity of AAP (**Table 3**).

**Table 3:** Comparison between mild/moderate and severe AAP groups in relation to the patients and disease characteristics, and outcome.

Variables	Mild/Moderate (N=47)	Severe (N=16)	P-value
Age (Years)			
<10	34 (72.3%)	5 (31.3%)	0.003
≥ 10	13 (27.7%)	11 (68.8%)	
Gender			
Male	29 (61.7%)	10 (62.5%)	1
Female	18 (38.3%)	6 (37.5%)	
Baseline WBC			
<50×10^9^/L	33 (70.2%)	10 (62.5%)	0.72
≥50×10^9^/L	14 (29.8%)	6 (37.5%)	
Immunophenotype			
B- Cell	35 (74.5%)	11 (68.8%)	0.75
T- Cell	12 (25.5%)	5 (31.3%)	
Treatment Regimen			
SR/HR	34(72.3%)	15 (93.8%)	0.2
LR	11 (23.4%)	1 (6.3%)	
VLR	2 (4.3%)	0 (0%)	
L- ASNase Dose Before AAP (IU/m^2^)			
Median [Min, Max]	45000 [5000, 282500]	56250 [15000, 242500]	0.176
L- ASNase injections Number Before AAP			
Median [Min, Max]	9.00 [1.00, 28.0]	9.00 [3.00, 26.0]	0.172
Admission to ICU			
No	47 (100%)	8 (50.0%)	<0.001
Yes	0 (0%)	8 (50.0%)	
AAP Outcome			
Recovery	47 (100%)	10 (62.5%)	<0.001
Death	0 (0%)	6 (37.5%)	

AAP, asparaginase- associated pancreatitis; WBC, white blood cell count; SR/HR, standard-risk/high-risk; LR, low-risk; VLR, very low-risk; L-ASNase, native E-coli asparaginase.

### 3.5 Clinical outcome and long-term sequelae

Approximately 90% of the patients (n=57) achieved clinical recovery from AAP, defined as the resolution of all symptoms, normalization of pancreatic enzyme levels, and restoration of pancreatic function, including improvements in nutritional and digestive status. The median time for clinical recovery was 8 days (range: 3–40 days). However, two patients developed persistent chronic pancreatic pseudocysts, and one patient developed diabetes mellitus, necessitating long-term insulin therapy following the occurrence of AAP. The mortality rate for patients with AAP was 9.5% (n=6), with all mortality cases occurring in the severe AAP group.

### 3.6 ASNase re-exposure

Asparaginase was reintroduced in 39 patients (62%) following complete clinical recovery, normalization of pancreatic enzyme levels, radiologic clearance of pancreatic inflammation and absence of complications such as necrosis or pseudocyst formation. Among these, 12 patients (30.8%) experienced recurrent AAP, with most recurrent episodes being mild to moderate, and only one patient suffered a severe recurrent episode resulting in acute pancreatic pseudocysts after a single ASNase dose. Notably, this patient’s first episode was likewise severe, presenting with hypovolemic shock. None of the patients with a second episode of AAP were further re-exposed to ASNase and there were no mortalities related to the second AAP episode. No significant differences were observed between patients with recurrent AAP and those without in relation to age, gender, SR/HR risk regimen, or severity of AAP first episode, however, the number of analyzed patients is small (**Table 4**).

**Table 4:** Risk factors affecting the recurrence of AAP.

Factors	Recurrence (n=12)	Non-Recurrence (n=27)	P- Value
Age (Years)			
<10	8 (66.7%)	18(66.7%)	0.65
≥ 10	4 (33.3%)	9 (33.3%)	
Gender			
Male	6 (50%)	18 (66.7%)	0.323
Female	6 (50%)	9 (33.3%)	
Treatment Regimen			
SR/HR	10 (83.3%)	22 (81.5%)	0.79
LR	2 (16.7%)	4 (14.8%)	
VLR	0 (0.0%)	1 (3.7%)	
AAP Initial Episode Severity			
Mild/Moderate	10 (83.3%)	26 (96.3%)	0.219
Severe	2 (16.7%)	1 (3.7%)	

AAP, asparaginase-associated pancreatitis; SR/HR, standard-risk/high-risk; LR, low-risk; VLR, very low-risk.

Two patients completed all the scheduled ASNase therapy before the onset of AAP, 26 patients resumed all the scheduled chemotherapy courses containing ASNase, and 26 patients continued their chemotherapy regimen without ASNase following an initial or recurrent episode of AAP. On the other hand, nine patients discontinued the chemotherapy protocol entirely, either due to deaths or hematopoietic stem cell transplantation.

Patients re-exposed to ASNase received a median of 18 additional doses (range 1–22) and a median of 22 total doses of ASNase (range 8–29) compared to a median of 15 doses (range 6–24) for those who were not re-challenged.

### 3.7 Primary disease outcome

**Figure 1:**
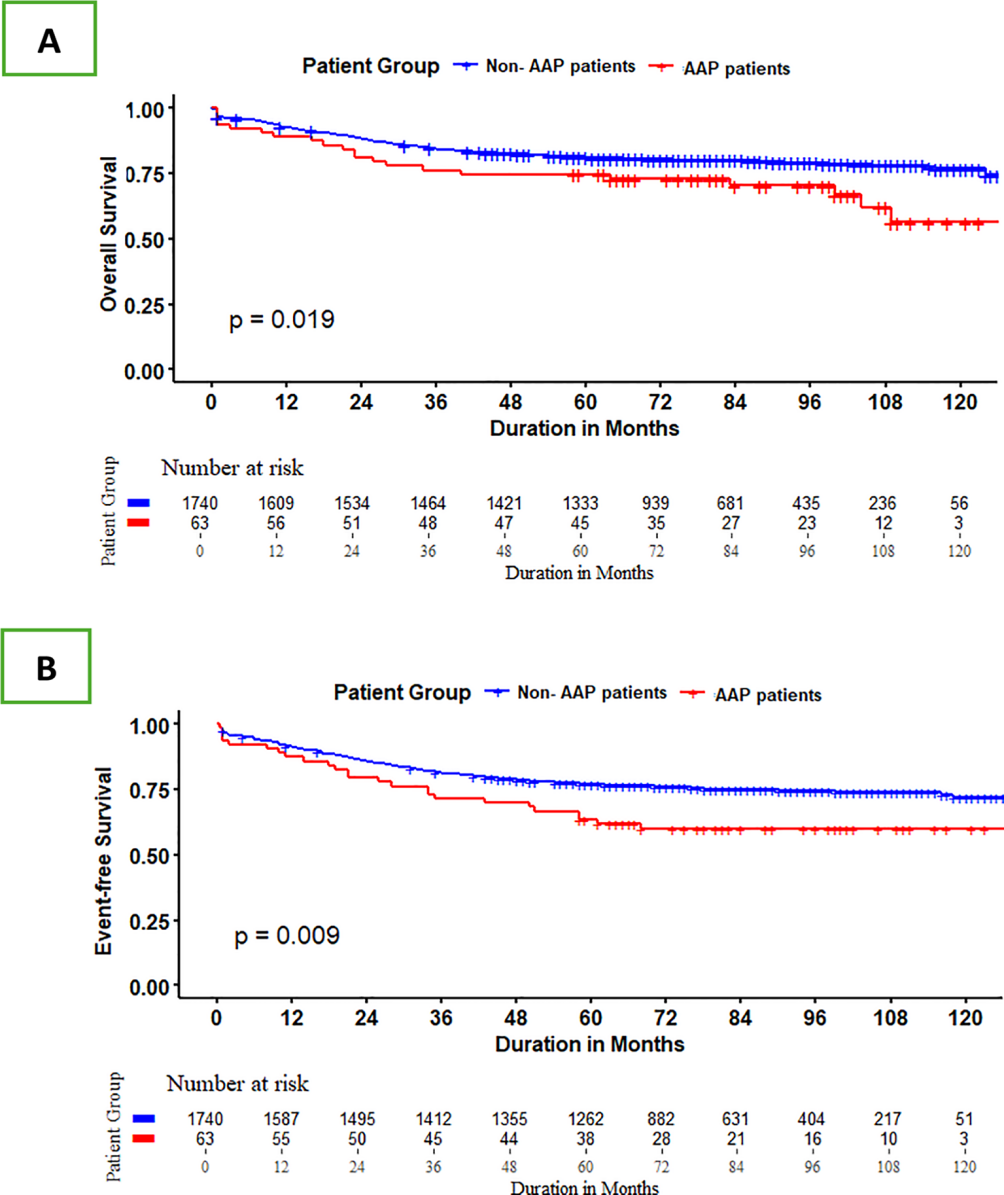
Five-year OS **(A)** and EFS **(B)** of patients with AAP compared to patients without AAP.

The median follow-up period for patients who remained alive was 83 months (range:16–136 months). Patients with AAP had a 5-year overall survival (OS) of 75% ± 5.48 compared to 81%+0.94% for those without (P= 0.02), and a 5-year event-free survival (EFS) of 63.5% ± 6% and 77% ± 1%, respectively (P=0.01) (**Figure 1**). However, in the multivariable Cox model, the significance of AAP was no longer observed after adjusting for other risk variables including patient age, immunophenotype, initial WBC, and ALL risk stratification. The adjusted hazard ratios were not statistically significant for OS (HR 1.27; 95% CI, 0.82-1.99; P=0.3) and EFS (HR1.32; 95% CI, 0.88-1.98; P=0.17) (**Supplementary Table S2**).

**Figure 2:**
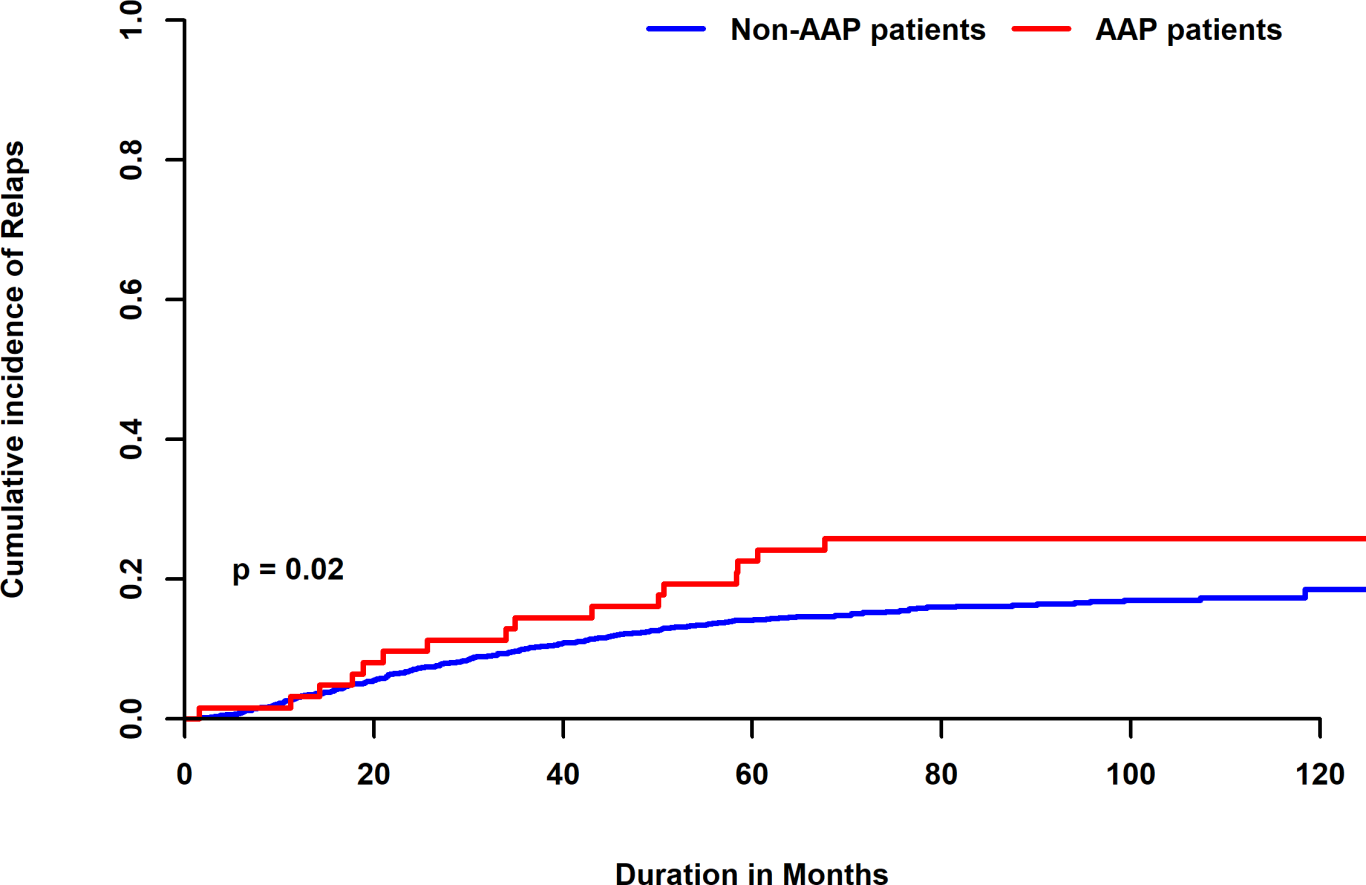
Five-year CIR of patients with AAP compared to patients without AAP.

Additionally, the 5-year cumulative incidence of relapse (CIR) was 24.3% in patients with AAP, which was statistically significantly higher than 14.4% reported in patients without AAP (P=0.02) (**Figure 2**). In multivariate analysis AAP lost its significance showing a relapse-specific hazard ratio (HR) of 1.44 (95% CI, 0.86-2.4; *P=0*.16) (**Supplementary Table S2**).

Patients who were not re-exposed to ASNase had a higher relapse rate (37.5%) compared to 23% for those who were re-challenged with ASNase.

## 4 Discussion

Asparaginase (ASNase) is a crucial drug in the treatment of pediatric ALL associated with improving survival rates. By reducing circulating asparagine levels, malignant lymphoblasts are targeted for apoptosis due to their limited ability to synthesize asparagine (2, 3). However, the use of ASNase is linked to substantial treatment-related toxicities, with AAP occurring in 2% to 18% of cases (18–22). This wide range of incidence may be due to variations in the intensity of ASNase regimens used across studies. In our cohort, the incidence of AAP among 1803 pediatric patients with ALL was 3.5%, consistent with the average rates reported in previous studies (21–23). The median interval between the last L-ASNase administration and the onset of AAP was 4 days. which was notably shorter than the reports from studies utilizing PEG-ASNase (24). This is attributed to the short plasma half-life of L-ASNase ranging from 8 to 30 hours compared to PEG-ASNase which have longer half-life of 5.8 days (6, 24).

The prevalence of AAP was at its highest during the remission induction phase aligning with previous studies which reported that more than half of AAP occurred during this phase (21–23).

Risk factors for the development of AAP have been previously widely investigated. Oparaji et al. (20) reviewed 10 studies, that met statistical criteria, from a pool of 1,842 screened articles and identified older age, asparaginase formulation, higher ALL risk stratification, and higher asparaginase dosing regimen as the most potential risk factors for the occurrence of AAP.

Similarly, studies by Samarasinghe (25), Barry (26), and Kearney (27) reported that older children and adolescents were more likely to develop this complication. Other studies have highlighted that a high-dose asparaginase regimen was the most reliable predictor of AAP (28). In our study, multivariate analysis identified age 10 years or older, and higher doses of ASNase regimens as independent risk factors for AAP.

In this context, it is crucial to recognize the significance of genetic susceptibility in the pathogenesis of AAP, which has received more emphasis in previous studies that have identified genes such as PRSS1, PRSS2, CTRC, SPINK1, CASR, CPA1, CLDN2, ASNS, CFTR, and ATF5 as potential predictors for AAP and severity (29–33). The lack of genetic analysis is a significant limitation in numerous studies including ours.

The incidence of severe AAP varies significantly, ranging from 7% to 86% (20, 21, 25). This considerable difference may be attributed to the use of different classification systems for grading the severity of acute pancreatitis (AP) across studies, including the CT severity index, Ponte di Legno Toxicity Working Group (PTWG), revised Atlanta classification, and CTCAE (2, 23, 25). Furthermore, intensity of chemotherapeutic regimens used, and variations in the asparaginase formulations’ half-life may have impacted the AAP severity in different studies. For instance, Liu et al. (23) found that nearly half of the patients in their study developed severe AAP. They linked this to the unique pharmacokinetics of PEG-ASNase, a formulation with a considerably longer half-life compared to native L- ASNase. Its enzymatic activity can persist for over 21 days, with serum asparagine depletion lasting between 26 and 34 days following a single dose. This prolonged activity may disrupt the balance of serum amino acids, leading to pancreatic damage and sustained toxic effects. In our study, the incidence of native L-ASNase severe AAP, equivalent to CTCAE Grade 4, was 25.3%. Patients ≥10 years demonstrated a higher susceptibility to severe AAP in previous studies and our findings confirmed this association (23, 34).

AAP is a serious and potentially life-threatening condition. It is associated with mortality and significant morbidity. Patients may experience conditions such as toxic pneumonia, acute respiratory distress syndrome, and renal failure which are the leading causes of mortality in severe AP cases. Although pseudocyst formation often resolves within weeks or months, infection can worsen the condition. Chronic pancreatitis with recurring abdominal pain is a long-term effect of AP and reactivation of chronic pancreatitis is a major cause of recurrent AP, even after discontinuation of ASNase (9, 15). The acute complications observed in our cohort were hypovolemic shock, MOF, acute necrotizing pancreatitis, and acute pancreatic pseudocysts, while long-term sequelae comprised chronic pancreatic pseudocysts along with persistently elevated pancreatic enzyme levels, and exocrine insufficiency requiring insulin therapy. The overall mortality associated with AAP was 9.5%, with all deaths occurring in patients with severe AAP. This rate is slightly higher than those reported in other studies (2, 21) and could be linked to associated severe infections and prolonged myelosuppression in some patients.

Given the well-established role of ASNase in treating pediatric ALL, the decision to truncate ASNase therapy due to toxicity must be carefully balanced against the potential risk of relapse. This consideration should account for the patient’s ALL risk stratification and the number of ASNase doses administered within the treatment protocol before the development of AAP.

Silverman et al. (12), showed that patients who had their ASNase therapy truncated due to toxicities and received less than 26 weeks out of 30 weeks of ASNase had a significantly worse outcome compared to those who completed at least 26 weeks of their scheduled ASNase weeks. Although patients in our study who developed AAP demonstrated significantly inferior 5-year EFS and an increased cumulative incidence of relapse, AAP was not identified as an independent risk factor for relapse. This may be attributed to the fact that some patients in the group who did not develop AAP might have other toxicities leading to truncation of ASNase doses or have lacked ASNase enzyme activity. In this respect, a study by the NOPHO group revealed a significantly increased risk of relapse among patients with truncated ASNase treatment or with no enzyme activity compared to those with measurable enzyme activity who were able to complete the full courses of ASNase therapy (35).

The incidence of AAP recurrence following re-exposure to ASNase varies considerably across the literature, with Knoderer et al. (10) reporting a recurrence rate of 7.7%, while Kearney et al. (27) documented a significantly higher rate of 62.5%. These discrepancies could be explained by varying criteria for ASNase reintroduction. Knoderer confined re-challenge to patients who had experienced mild AAP with complete symptoms recovery within 72 hours, whereas Kearney required only resolution of symptoms without a focus on AAP episode severity.

About 30% of the patients in our cohort who were re-challenged with ASNase experienced a second episode of pancreatitis. Notably, most of these recurrent episodes were mild to moderate in severity and not associated with any mortality. Furthermore, these patients received a median of 18 additional doses of ASNase, suggesting that additional doses after an initial AAP episode recovery may be feasible in pediatric patients.

Our clinical approach has been to re-introduce ASNase in patients who experienced relatively mild to moderate AAP, patients who developed pancreatic necrosis or pseudocysts during their initial episode of pancreatitis, were not eligible for asparaginase re-challenge. In our series, 39 patients (62%) were reintroduced to asparaginase after recovering from AAP. This approach may differ from other studies that decided to truncate ASNase due to concerns about the likelihood of AAP recurrence (25, 34).

Efforts failed to identify risk factors associated with AAP recurrence upon re-exposure to ASNase, mostly due to the small number of these patients in our study as in previous studies (2, 21). Nielsen and colleagues developed machine learning models that introduce age, gender, pancreatitis gene polymorphisms, and other risk variables to assess the likelihood of AAP recurrence. Implementation of this approach may offer an objective tool for determining the recurrence risk of AAP (36).

Our study’s limitations include its retrospective nature, not considering other causes that might have led to the omission of ASNase doses in the other group of patients without AAP, the lack of therapeutic drug monitoring of ASNase activity, and the lack of genetic susceptibility testing. Despite the large overall sample of 1803 patients, only 63 developed acute pancreatitis; consequently, even with methods to reduce sparse‐data bias, the adjusted hazard‐ratio estimates for pancreatitis in our multivariate survival models are based on a limited number of events and should be interpreted with caution.

In conclusion, the incidence of AAP among pediatric ALL patients was 3.5%. Older age at diagnosis and high doses of ASNase regimen were identified to be independent risk factors for developing AAP. Patients with AAP had a higher risk of relapse than those without. Notably, 30.8% of the patients experienced a recurrence of AAP upon re-exposure to ASNase which were mostly mild to moderate in severity. The decision to reintroduce ASNase must be carefully evaluated, balancing the risk of pancreatitis recurrence against leukemia relapse. Future research may consider exploring genetic susceptibility for pancreatitis and implementing machine learning models to identify patients at risk of AAP.

## Data Availability

The original contributions presented in the study are included in the article/**Supplementary Material**. Further inquiries can be directed to the corresponding authors.
