# Supplementary material for: Risk factors and outcome of asparaginase-associated pancreatitis in pediatric acute lymphoblastic leukemia
Source: Front Oncol. 2025 Jun 26;15:1606261. doi: 10.3389/fonc.2025.1606261 (PMC12241112; doi:10.3389/fonc.2025.1606261)
Supplement: Supplementary file 1 [file DataSheet1.pdf]

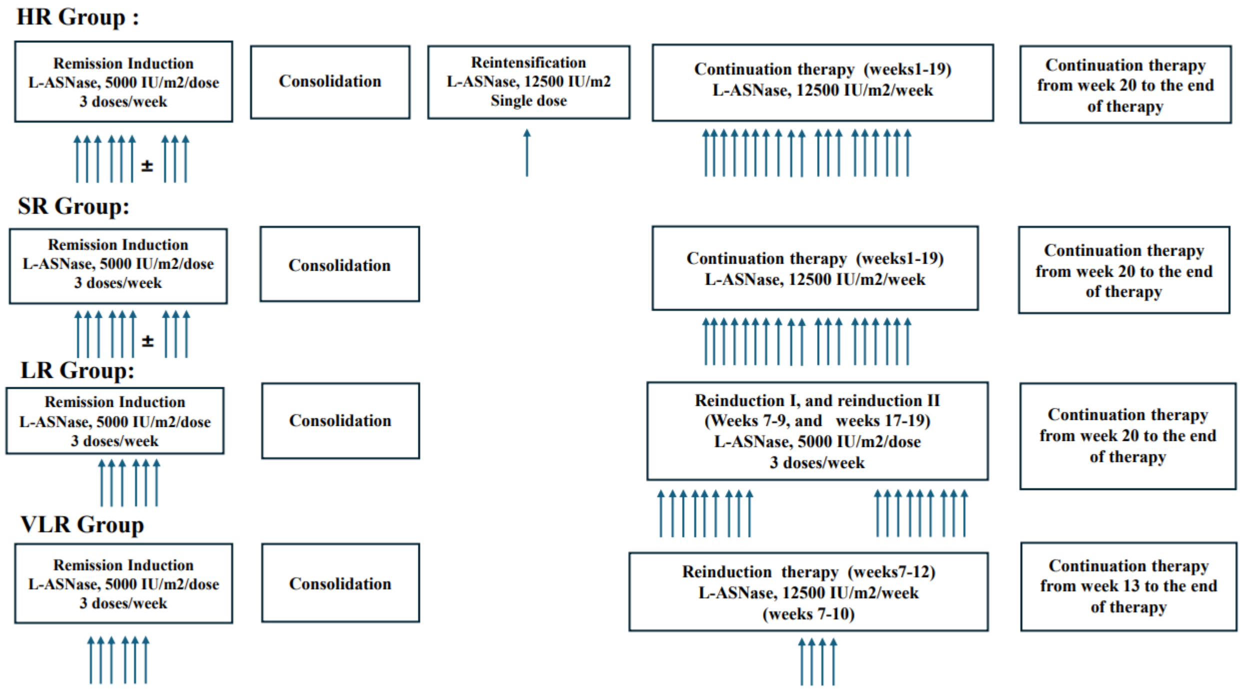

Figure S1: Outline of CCHE-Adopted St. Jude ALL Total XV treatment regimen. Arrows indicate the timings of L-Asparaginase (Medac- ASNase).

Table S1: Acute Lymphoblastic leukemia Risk stratification.

Very Low Risk	Low Risk	Standard Risk	High Risk
Precursor B-cell ALL with:<br>-Age:1year- <10 years<br>- WBC:<$50x10^9$ /L<br>- CNS1[*1] or CNSII[*2]<br>- MRD day 15 remission induction <0.01% and<br>-MRD end of remission induction <0.01%.<br>- Without unfavorable features[*4,] and [*5] Adverse genetic features	Precursor B-cell ALL with:<br>-Age: ≥10 years OR WBC ≥$50x10^9$ /L with DNA index ≥ 1.16 OR t(12,21)<br><br>**OR**<br>- Age: 1year- <10 years & WBC <$50x10^9$ /L<br><br>- CNS1[*1] or CNSII[*2]<br>- MRD day15 remission induction <1% and MRD end of remission induction <0.01%<br>- Without unfavorable features[*4] and [*5] Adverse genetic features	1-All T-cell of ALL<br>2- Precursor B-cell ALL with:<br>-Age: ≥10 years<br>-OR WBC ≥ $50x10^9$ /L<br>-OR CNS III[*3]<br>_OR Overt testicular leukemia by U/S.<br>-OR Adverse genetic features as: -t(1,19)<br>-MLL gene rearrangement on (11q23)<br>- OR poor early response:<br>MRD ≥1 at day15 remission induction and /OR<br>MRD (≥0.01% and <1) at end of remission induction	BCR ABL fusion gene: t(9; 22)<br>-OR t(17,19)<br>-OR (iAMP21) [*6]<br>- OR Hypodiploidy (<44 chromosomes) by karyotyping.<br>-OR ALL patients with MRD ≥1% by FCM at end of induction.<br>- OR ALL patients with MRD ≥0.1% by FCM on week 7 continuation therapy.

-[*1] CNS I: (< 5 WBC/L of CSF without blasts). -[*2] CNS II: (< 5 WBC/L of CSF with blasts).

- [*3]CNS III Status: (≥ 5 WBC/L of CSF with blast or cranial nerve palsy).

-[*4] Unfavorable Features: Overt testicular leukemia by U/S, CNS III.

-[*5] Adverse genetic features: a) t(1;19), b) t(17;19), c) MLL gene rearrangement (11q23), d) BCR-ABL fusion gene t(9, 22), e) Hypodiploidy: (<44 chromosomes), and f) [*6] Intra-chromosomal amplification of chromosome 21(iAMP21), defined as: having three or more RUNX1 signals on a single chromosome 21, or a total of five or more RUNX1 signals per cell.

Table S2: Multivariate analysis of prognostic factors in relation to OS, EFS and CIR in pediatric ALL Patients

	OS		EFS		CIR	
**Risk Variables**	**Odds ratio (95% CI)**	**P-value**	**Odds ratio (95% CI)**	**P-value**	**Odds ratio (95% CI)**	**P-value**
**AAP**						
No (ref)						
Yes	**1.27 (0.82-1.99)**	**0.3**	**1.32 (0.88-1.98)**	**0.17**	**1.44 (0.86-2.4)**	**0.16**
**Age (Years)**						
<10 (Ref)						
≥ 10	**1.21 (0.95-1.51)**	**0.1**	**1.21 (0.98-1.51)**	**0.07**	**1.28 (0.96-1.69)**	**0.085**
**Risk Stratification**						
SR(Ref)						
HR	**2.13 (1.62-2.81)**	**<0.001**	**2.16 (1.67-2.79)**	**<0.001**	**2.44 (1.74-3.42)**	**<0.001**
LR	**0.41 (0.30-0.56)**	**<0.001**	**0.51 (0.39-0.67)**	**<0.001**	**0.66 (0.47-0.92)**	**0.014**
VLR	**0.27 (0.16-0.44)**	**<0.001**	**0.37 (0.25-0.56)**	**<0.001**	**0.49(0.31- 0.79)**	**0.003**
**Immunophenotype**						
B- Cell (Ref)						
T- Cell	**1.14 (0.88-1.47)**	**0.3**	**1.06 (0.84-1.35)**	**0.6**	**0.89 (0.64-1.23)**	**0.49**
**Baseline WBC**						
$<50\times10^9$ /L (Ref)						
$\geq50\times10^9$ /L	**1.06 (0.83-1.35)**	**0.6**	**1.18 (0.95-1.47)**	**0.13**	**1.46 (1.11-1.91)**	**0.006**

OS: overall survival; EFS: event-free-survival; CIR: cumulative incidence of relapse; AAP: asparaginase-associated pancreatitis; WBC: white blood cell count; SR/HR: standard-risk/high-risk; LR: low-risk; VLR: very low-risk
